# Synthesis and Characterization of Novel Cu(II), Pd(II) and Pt(II) Complexes with 8-Ethyl-2-hydroxytricyclo(7.3.1.0^2,7^)tridecan-13-one-thiosemicarbazone: Antimicrobial and *in Vitro* Antiproliferative Activity

**DOI:** 10.3390/molecules21050674

**Published:** 2016-05-21

**Authors:** Elena Pahonțu, Codruța Paraschivescu, Diana-Carolina Ilieș, Donald Poirier, Camelia Oprean, Virgil Păunescu, Aurelian Gulea, Tudor Roșu, Ovidiu Bratu

**Affiliations:** 1 Inorganic Chemistry Department, Faculty of Pharmacy, University of Medicine and Pharmacy ”Carol Davila”, 6 Traian Vuia Street, Bucharest 020956, Romania; 2 Organic Chemistry Department, Faculty of Chemistry, University of Bucharest, 90-92 Panduri Street, Bucharest 050663, Romania; 3 Organic Chemistry Department, Faculty of Pharmacy, University of Medicine and Pharmacy ”Carol Davila”, 6 Traian Vuia Street, Bucharest 020956, Romania; ilies_diana@hotmail.com; 4 Oncology and Molecular Endocrinology Research Center CHUL, Research Center and Universite Laval, CHUQ-CHUL, 2705 Boulevard Laurier, Quebec City, QC G1V 4G2, Canada; donald.poirier@crchul.ulaval.ca; 5 Pharmaceutical Chemistry Department, Faculty of Pharmacy, University of Medicine and Pharmacy “Victor Babeş”, Timişoara 300041, Romania; camelia.sass5@gmail.com; 6 Functional Sciences Department, Faculty of Medicine, University of Medicine and Pharmacy “Victor Babeş”, 2 Eftimie Murgu Square, Timişoara 300041, Romania; genostem@yahoo.com; 7 Coordination Chemistry Department, Moldova State University, 60 Mateevici Street, Chisinau 2009, Moldova; dociu1946@yahoo.com; 8 Inorganic Chemistry Department, Faculty of Chemistry, University of Bucharest, 23 Dumbrava Rosie Street, Bucharest 020462, Romania; t_rosu0101@yahoo.com; 9 Clinic of Urology, Universitary Emergency Central Military Hospital, Clinical Departament 3, University of Medicine and Pharmacy “Carol Davila”, 6 Traian Vuia Street, Bucharest 020956, Romania; ovi78doc@yahoo.com

**Keywords:** copper(II), palladium(II) and platinum(II) complexes, antimicrobial activity, HL-60 leukemia cells, NCI-H1573 lung adenocarcinoma cells, SKBR-3 human breast cancer cells, MCF-7, A375 human melanoma cells

## Abstract

New Cu(II), Pd(II) and Pt(II) complexes, (Cu(L)(H_2_O)_2_(OAc)) (**1**), (Cu(HL)(H_2_O)_2_(SO_4_)) (**2**), (Cu(L)(H_2_O)_2_(NO_3_)) (**3**), (Cu(L)(H_2_O)_2_(ClO_4_)) (**4**), (Cu(L)_2_(H_2_O)_2_) (**5**), (Pd(L)(OAc))H_2_O (**6**), and (Pt(L)_2_) (**7**) were synthesized from 8-ethyl-2-hydroxytricyclo(7.3.1.0^2,7^)tridecan-13-one thiosemicarbazone (**HL**). The ligand and its metal complexes were characterized by IR, ^1^H-NMR, ^13^C-NMR, UV-Vis, FAB, EPR, mass spectroscopy, elemental and thermal analysis, magnetic susceptibility measurements and molar electric conductivity. The free ligand and the metal complexes have been tested for their antimicrobial activity against *E. coli*, *S. enteritidis*, *S. aureus*, *E. faecalis, C. albicans* and cytotoxicity against the NCI-H1573 lung adenocarcinoma, SKBR-3 human breast, MCF-7 human breast, A375 human melanoma and HL-60 human promyelocytic leukemia cell lines. Copper complex **2** exhibited the best antiproliferative activities against MCF-7 human breast cancer cells. A significant inhibition of malignant HL-60 cell growth was observed for copper complex **2**, palladium complex **6** and platinum complex **7**, with IC_50_ values of 1.6 µM, 6.5 µM and 6.4 µM, respectively.

## 1. Introduction

Thiosemicarbazones represents a class of *N*,*S*-donor ligands important in coordination chemistry. These ligands have great versatility, manifested by the existence of two forms (thione-thiol), and the ability to bind metal ions in the neutral or anionic form, acting as monodentate or bidentate ligands [1,2,3,4,5,6]. Metal thiosemicarbazone complexes are of interest due their bioinorganic applications. Research in recent years has demonstrated the ability of thiosemicarbazones and their complexes to be antifungal, antibacterial, antiviral, anti-inflammatory and chemotherapeutic agents, potentially useful for inhibiting the activities of cancer cells [7,8,9,10,11,12,13,14,15,16,17]. The cytotoxicity of these ligands is enhanced by coordination to metal ions such as copper, zinc, platinum and palladium. This activity is explained not only by the metals’ ability to influence lipophilicity but also the mechanism of action within the cell [18,19,20,21,22,23,24,25,26]. Substitution on the C^2^ position can also affect the coordination and biological properties. The introduction of different groups at the C^2^ position was made to vary the hydrophilic-lipophilic character of the compounds in order to increase the cell absorption [27].

In a previous paper [28] we presented the synthesis of Cu(II) and Pd(II) complexes with 8-propyl-2-hydroxytricyclo(7.3.1.0^2,7^)tridecan-13-one-thiosemicarbazone and 8-furyl-2-hydroxytricyclo(7.3.1.0^2,7^)tridecan-13-one-thiosemicarbazone. This paper is a continuation of our previous research and it presents the synthesis and characterization of complexes of Cu(II), Pd(II) and Pt(II) with the new ligand 8-ethyl-2-hydroxytricyclo(7.3.1.0^2,7^)tridecan-13-one-thiosemicarbazone, a less voluminous substituent. The anti-proliferative activity of the ligand and its synthesized complexes in NCI-H1573 lung adenocarcinoma, SKBR-3 human breast, MCF-7 human breast, A375 human melanoma cancer cells and human HL-60 promyelocytic leukemia cells were investigated. The complexes and ligand were also tested for their *in vitro* antibacterial activity against *Escherichia coli*, *Salmonella enteritidis*, *Staphylococcus aureus, Enterococcus* *faecalis* and *Candida albicans* strains using the paper disc diffusion method [29] (for the qualitative determination) and the serial dilutions in liquid broth method [30] (for determination of MIC).

## 2. Results and Discussion

### 2.1. Chemistry

**Scheme 1:**
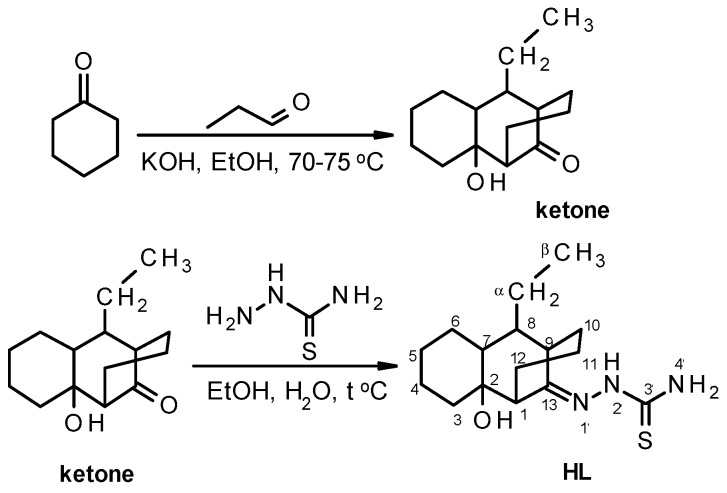
Synthesis of the ligand 8-ethyl-2-hydroxytricyclo(7.3.1.0^2,7^)tridecan-13-one-thio-semicarbazone (**HL**).

The tiosemicarbazone **HL** was synthesized by refluxing equimolar amounts of thiosemicarbazide and β-cyclocetol in methanol according to the experimental protocol described in [28] (Scheme 1). The structure of the ligand was established by IR, ^1^H-NMR, ^13^C-NMR and mass spectroscopy data.

For the preparation of complexes **1**–**7** were used the metal salts Cu(OAc)_2_·(H_2_O), CuSO_4_·5H_2_O, Cu(NO_3_)_2_·3 H_2_O, Cu(ClO_4_)_2_·6 H_2_O, CuCl_2_·2H_2_O, Pd(OAc)_2_ and K_2_(PtCl_4_) which were refluxed with stirring in a hot clear solution of the ligand in methanol (1:1 and 1:2 metal–ligand ratio). All complexes are microcrystalline solids, which are stable in air. The melting point values are greater than 220 °C. The complexes are insoluble in organic solvents such as methanol, ethanol, chloroform and acetone, but soluble in DMF and DMSO.

**Figure 1:**
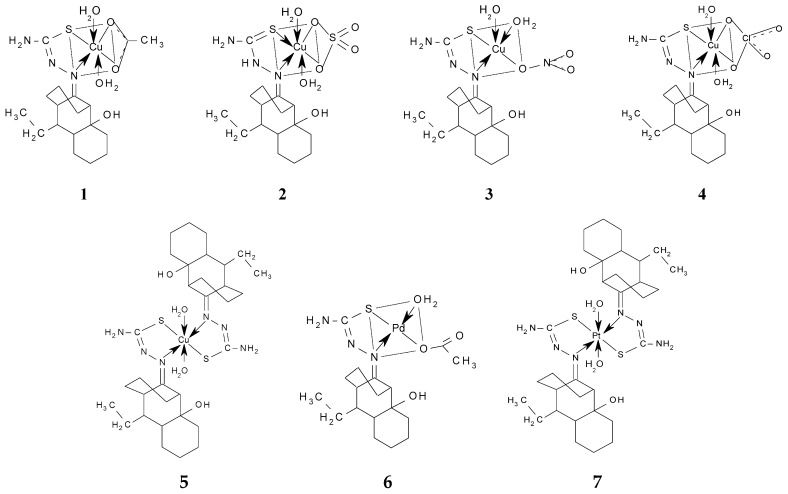
Proposed structures of the newly obtained metal complexes **1**–**7***.*

The proposed molecular formulas for thiosemicarbazone **HL** and complexes **1**–**7** (Figure 1) are in agreement with the stoichiometries obtained based on analytical data (see Experimental). The molar conductance values of the soluble complexes in DMF (8–15 ohm^−1^∙cm^2^∙mol^−1^) showed that all complexes are non-electrolytes [31].

#### 2.1.1. ^1^H-NMR and ^13^C-NMR Spectra

The ^1^H-NMR (DMSO-*d*_6_) spectra of diamagnetic Pd(II) and Pt(II) complexes showed the disappearance of the signal due to the =N-NH proton, indicating the deprotonation of this group, while the signals of the other NH (NH_2_) protons were observed at 10.42 and 10.44 ppm, respectively. The signal due to the OH proton remained unchanged as observed in the free ligand. In the ^13^C-NMR (DMSO-*d*_6_) spectra of the two complexes was observed that imine C=N carbon atoms signal is shifted downfield and appeared at 173.274 and 175.208 ppm, respectively. This information indicates the coordination of the metal center to the azomethine nitrogen and the sulfur in the thiol form and the NH_2_ group is not coordinated to the metal ion [32].

#### 2.1.2. Infrared Spectra and Coordination Mode

The thiosemicarbazone group can exhibit thione-thiol tautomerism, because it contains a thioamide functional group. The presence of the band υ(N^2′-^H) at 3214 cm^−1^ and the absence of the one from 2570 cm^−1^ specific to υ(SH), indicates the ligand’s existence in thione form in a solid state [33]. In all IR spectra of the complexes the band at 3214 cm^−1^ is shifted to lower wavenumbers by 38 cm^−1^ and the presence of new band at 1617–1631 cm^−1^ (except for **2**), due to the υ(N^2′^=C) group, indicates the C=S thioenolisation followed by deprotonation and complexation with the metal ions. The band at 3360 attributed to the υ(N^4′^-H) stretching mode in the spectra of the ligand did not present any considerable change in the complexes and thus ruled out the possibility of metal ion bonding in this area [34]. Also, in the spectrum of ligand **HL** two bands appear at 1276, 815 cm^−1^ assigned to the υ(C=S) + υ(C=N) frequencies, respectively, and the υ(C=S) (tioamide IV) frequency. In the complexes’ spectra these bands are shifted towards lower frequencies indicating the coordination of the thione/thiol sulphur [35,36]. On the other hand, in the spectra of all complexes (except **2**) the emergence of a new band in the 581–628 cm^−1^ range, due to υ(C-S) vibration is also a sign of sulphur coordination in the thiol form.

The presence of υ_as_(COO^−^) and υ_s_(COO^−^) absorption bands at 1648, 1533 cm^−1^ and 1565, 1473 cm^−1^, respectively, in the IR spectra of complexes **1** and **6** suggests that bidentate behavior of the acetate group [37].

Three bands, which appeared at 1211, 1118, 1089 cm^−1^ in the IR spectrum of complex **2** are assignable to ʋ_3_ of the bidentate coordinated sulfato group. Further, the presence of the medium bands (ʋ_1_) at 931 cm^−1^ confirm the bidentate behaviour of the sulfato group [38].

The nitrate complex **3** has two bands at 1404 and 1210 cm^−1^ corresponding to υ_5_ and υ_1_, with a separation of 194 cm^−1^, and a medium band at 934 cm^−1^ assigned to υ_2_ of the nitrate group. The separation υ_5_–υ_1_ can be used as a criterion to distinguish between the degree of covalence of the nitrate coordination. These values indicate the presence of a terminal monodentate nitrate group [39].

The perchlorate complex **4** shows a band at 1089 cm^−1^ split into three components and a strong band at 628 cm^−1^. Splitting of this band indicates a bidentate coordination of the perchlorate group [38].

The frequency at 3436 cm^−1^ in **HL**, is assigned to the stretching vibration of the OH group of the thiosemicarbazone nucleus. All the complexes (except **7**) exhibit υ(OH) and γ(H_2_O) bands in the 3445–3453 and 1132–1143 cm^−1^ regions, which are indicative of coordinated water [40].

#### 2.1.3. Electronic Spectra and Magnetic Studies

The electronic spectrum in the polycrystalline state of ligand **HL** showed the maximum intraligand absorption: 34,820 and 30,900 cm^−1^, assigned to π→π* and n→π* transitions, corresponding to azomethine and thioamide groups of the ligand [41]. These bands are shifted after complexation and new bands appeared in the 28,250–27,340 cm^−1^ region and were attributed to S→Cu(II) charge transfer bands.

The electronic spectra for complexes **1**, **2**, **4** and **5** show d–d bands in the regions 12,550–13,570 and at 20,000 cm^−1^ (Figure S1) which may be assigned to $d_{x^{2}-y^{2}}$→$d_{xy}$ and $d_{x^{2}-y^{2}}$→$d_{xz,yz}$ transitions, respectively, due to the distorted octahedral geometry (D_4h_) around Cu(II) [42].

Likewise, the electronic spectra of the Cu(II) complex **3** suggests the typical axial behavior for a square-pyramidal geometry with a $d_{x^{2}-y^{2}\;}$ ground state [43].

The electronic spectra of the complexes **6** and **7** showed a square-planar geometry for the palladium and platinum ions. The very intense bands at about 22,220 cm^−1^ and 23,250 cm^−1^, respectively, are assignable to a combination of metal–ligand charge transfer and d–d bands (Table 1) [44].

The values of µ_eff_, for complexes **1**–**5** suggest the presence of an unpaired electron. They also indicate the existence of monomeric species.

#### 2.1.4. Mass Spectra

The FAB mass spectra of the Cu(II) complexes with thiosemicarbazone **HL** have been recorded (Table S1). The molecular ion [M]^+^ peaks obtained from complexes are as follow: *m*/*z* = 372.5 (**1**), *m*/*z* = 374.2 (**2**), *m*/*z* = 371.5 (**3**), *m*/*z* = 372.5 (**4**), *m*/*z* = 681.3 (**5**), *m*/*z* = 474.5 (**6**), *m*/*z* = 794.1 (**7**). The data obtained are in good agreement with the proposed molecular formulae for complexes **1**–**7**. The FAB mass spectra of these complexes show peaks assignable to various fragments arising from the thermal cleavage of the complexes.

#### 2.1.5. Thermal Decomposition

The thermal decompositions of the complexes **1**–**7** were studied by thermogravimetry (TG). For complexes **1**–**6** the thermogravimetric analysis show a weight loss in the range 138.89–170.03 °C. This mass loss corresponds to the elimination of two water molecules (in **1**–**5**) and one molecule of water for complex **6**, respectively. The second weight loss steps correspond to the release of small coordinated anions: OAc^−^, SO_4_^2−^, NO_3_^−^ (complexes **1**–**3** and **6**). The other weight loss refers to the decomposition of the ligand. The final residue for complexes **1**–**3** and **5** was analyzed by IR spectroscopy, which confirmed the formation of CuO (Figure S2).

#### 2.1.6. EPR Spectra

**Figure 2:**
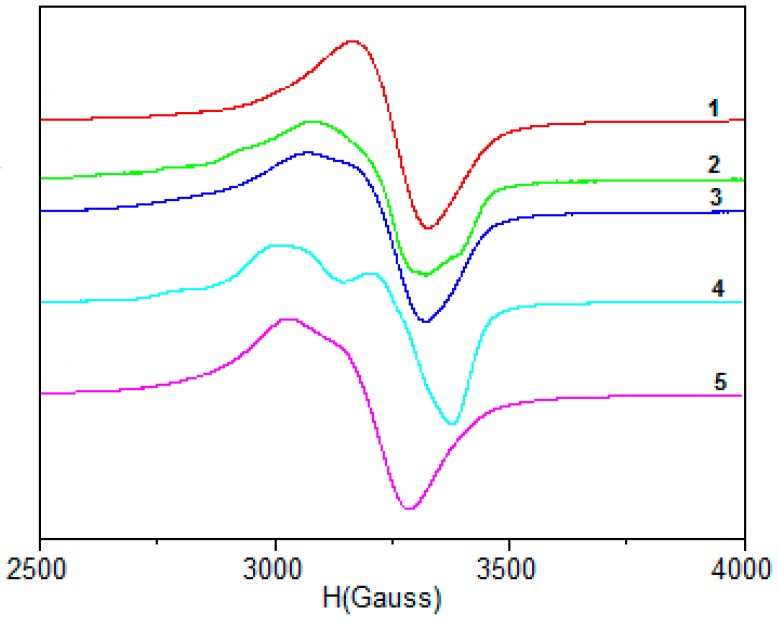
EPR spectra of complexes **1**–**5** in the polycrystalline state at 298 K.

To elucidate the structure of the copper complexes, EPR spectra were recorded in the polycrystalline state and in frozen DMSO solution. The EPR spectral assignments of the copper complexes **1**–**5** and orbital reduction parameters in the polycrystalline state at 298 K and in DMSO solution at 298 K and 77 K are presented in Table 2. The EPR spectra of the complexes recorded in the polycrystalline and solution state provide information about the coordination environment around Cu(II). The spectrum of the complex **1** in the polycrystalline state at 298 K shows a signal consisting of a single slightly asymmetrical broad line, with ΔH = 160G (Figure 2).

The EPR spectrum of complex **2** shows a signal with a breadth of approximate 260 G and parameters A_//_ = 135 G, g_//_ = 2.371 and g_⊥_ = 2.103.

**Figure 3:**
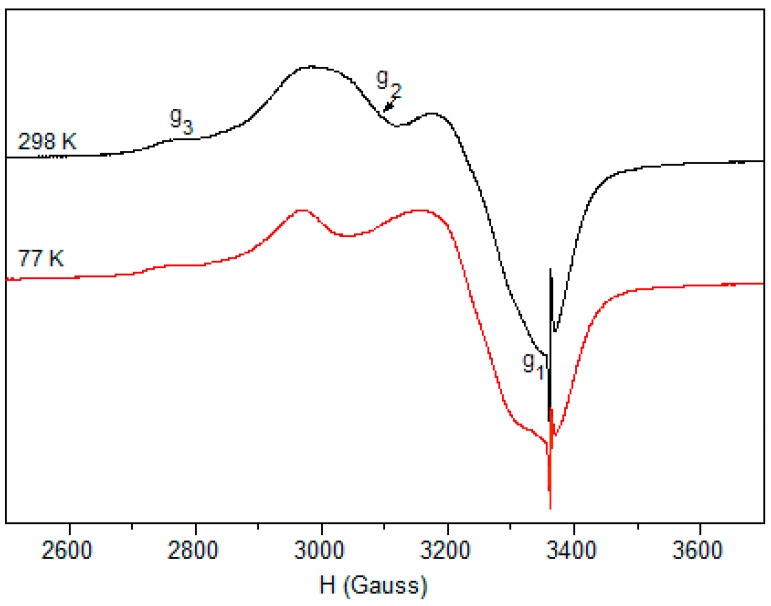
EPR spectrum of complex **5** in the state at 298 K and 77 K.

The spectra of the complexes **3** and **5** show axial symmetry with well-defined g_//_ and g_⊥_ values, respectively g_//_ = 2.208; g_⊥_ = 2.041 (for **3**) and g_//_ = 2.237; g_⊥_ = 2.065 (for **5**). In these Cu(II) complexes, tensor values of g_//_ > g_⊥_ > 2.0023 are consistent with a d_x_^2^_−y_^2^ ground state [45]. The EPR spectrum of complex **4** (Figure 3) both at room temperature (298 K) as well as at 77 K, is typical for an orthorhombic symmetry center, for which the evaluated parameters are: g_1_ = 2.011; g_2_ = 2.193; g_3_ = 2.409.

To assess the exchange interaction between the copper centers in the polycrystalline compound is calculated geometric parameter G using the equation G = (g_//_ − 2.0023)/(g_⊥_ − 2.0023) for axial spectra [46]. If G is higher than 4, then the exchange interaction is negligible (compounds **1**–**3**, **5**), but if G is less than 4, considerable exchange interaction is indicated in the solid complex (for complex **4**).

**Figure 4:**
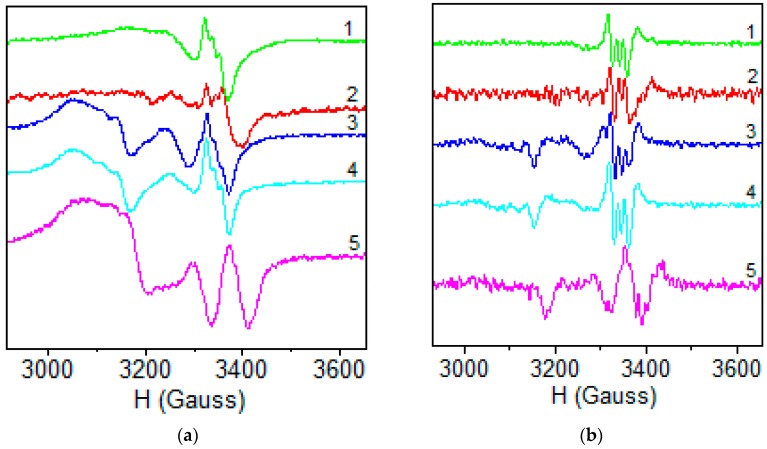
EPR spectra of complexes **1**–**5** in DMSO solution (**a**) at 298 K; (**b**) second derivative spectra.

The EPR spectrum of complex **5** recorded in DMSO solution at 298 K and its second derivative, which shows five nitrogen superhyperfine lines in the high field component are given in Figure 4a,b. For complexes **1**–**4**, three nitrogen superhyperfine lines in the high field component are observed.

**Figure 5:**
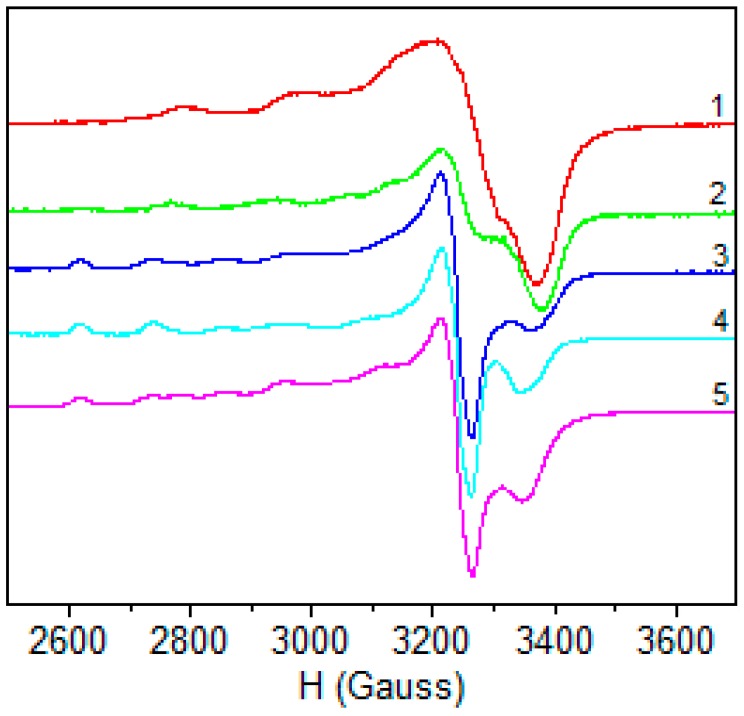
EPR spectra of complexes **1**–**5** in DMSO solution at 77 K.

The spectra of complexes **1**, **2**, **4** and **5**, in DMSO solution at 77 K, show a typical distorted octahedral geometry and hyperfine splitting into four lines (Figure 5). These show four well defined hyperfine lines in the parallel region corresponding to the electron spin–nuclear spin interaction. In all Cu(II) complexes tensor values of g_//_ > g_⊥_ > 2.0023 are consistent with a d_x_^2^_−y_^2^ ground state [47].

The EPR parameters g_//_, g_⊥_, A_//_ and the energies of the d–d transition were used to evaluate the bonding parameters α^2^, β^2^ and δ^2^, which may be regarded as measures of the covalency of the in-plane σ bonds, in-plane π bonds and out-of-plane π bonds [48]. The orbital reduction factors K_//_ = α^2^β^2^ and K_⊥_ = α^2^δ^2^, were calculated using expressions reported elsewhere [49]. Hathaway has pointed out that for pure σ bonding, K_//_ ≈ K_⊥_ ≈ 0.77 and for in-plane π-bonding, K_//_ < K_⊥_, while for out-of-plane π-bonding, K_//_ > K_⊥_ [46].

The empirical factor f = g_//_ /A_//_ cm^−1^ is an index of tetragonal distortion. The values of this factor may vary from 105 to 135 for small to extreme distortions in square planar complexes and depends on the nature of the coordinated atoms [39,50]. The f values of complexes **1**–**5** are higher than 135, indicating significant distortion from planarity.

### 2.2. Antibacterial and Antifungal Activity

All synthesized complexes and ligand were tested for their *in vitro* antibacterial activity against Gram-positive bacteria (*S. aureus*, *E. faecalis*), Gram-negative bacteria (*E. coli*, *S. enteritidis*) and anti-fungal activity (*C. albicans*) strains using paper disc diffusion technique (for the qualitative determination) and the serial dilutions in liquid broth method (for determination of MIC). Furacillinum and nystatin were used as standard drugs.

The results of the antimicrobial activity study of the synthesized compounds are shown in Table 3. It shows that the ligand **HL** type thiosemicarbazones show only bacteriostatic activity against the microorganisms named above, concentration limits 0.5–10 mg/mL, and coordination compounds of copper with this ligand have both bacteriostatic and bactericidal activity in the concentration range 0.12–10 mg/mL for the Gram-positive bacteria and 0.25–10 mg/mL against Gram-negative bacteria.

These experimental data shows that copper ion coordination of the thiosemicarbazone favors an increased antimicrobial activity. In many cases, the minimum inhibitory concentrations (MIC) of the synthesized compounds are close to or coincide with the minimum bactericidal concentrations, which confirms the bactericidal antimicrobial activity of these coordination compounds. The experimental data show that the highest sensitivity to the investigated compounds corresponds to Gram-positive (MIC = 0.12–0.5 mg/mL, MBC = 0.12–10 mg/mL) bacteria and *Candida albicans* (MIC = 0.12–10 mg/mL, CMB = 0.12–10 mg/mL), and the highest resistance is manifest in Gram-negative (MIC = 0.25–10 mg/mL, MBC > 10 mg/mL) strains. It may be observed that the antibacterial and antifungal activity of the coordination compounds is directly increased by the amount of thiosemicarbazone **HL**, the internal area and the nature of the acid to ligand bond. The detected properties of the synthesized compounds are of interest in terms of extending antibacterial and antifungal arsenal of existing drugs.

### 2.3. Antiproliferative Activity

**Figure 6:**
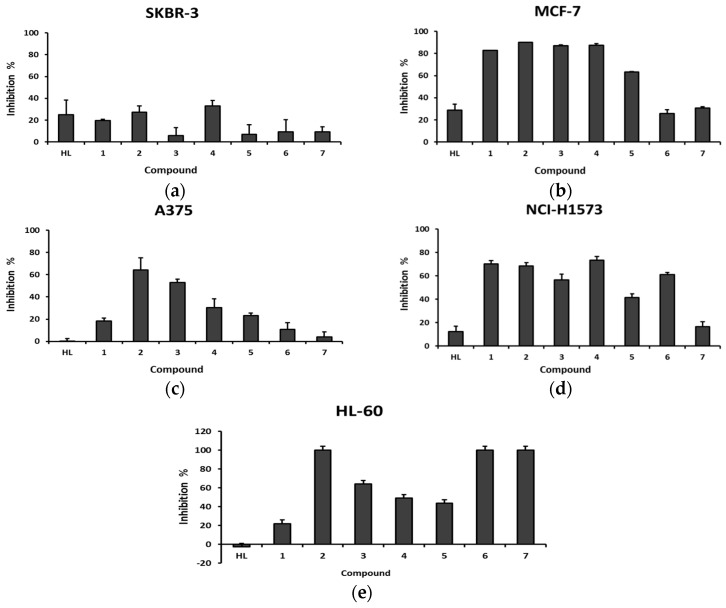
The effects of ligand **HL** and complexes **1**–**7** on SKBR-3, MCF-7, A375, NCI-H1573 and HL-60 cell proliferation. (**a**) Inhibition of cell proliferation on SKBR-3 human breast cancer cell line; (**b**) Inhibition of cell proliferation on MCF-7 human breast cancer cell line; (**c**) Inhibition of cell proliferation on A375 melanoma cell line; (**d**) Inhibition of cell proliferation on NCI-H1573 lung adenocarcinoma cell line; (**e**) Inhibition of cell proliferation on human leukemia HL-60 cells after exposure to 10 µM of tested compounds.

Using the Alamar blue or MTS antiproliferative assays, the antiproliferative activity of **HL** and complexes on several primary cancer cell lines (A375, SKBR-3, MCF-7 and NCI-H1573) and on HL-60 leukemia cells was tested. Figure 6 show the percent inhibition of the compounds on the tested cell lines after 48 h of exposure.

In the case of primary cancer cell lines, one can notice (Table S2) that for all the compounds the highest antiproliferative activity was for the NCI-H1573 lung adenocarcinoma cell line, while the SKBR-3 breast cancer cell line seems to be the most resistant to treatment. The ligand **HL** proved to exert a moderate antiproliferative activity on breast cancer, with an inhibition index of 28.57% and 24.87% for MCF-7 and SKBR-3 cells, respectively.

A lack of **HL** activity was detected for A375 cells. In the case of NCI-H157 cells, the inhibition was 12.37%. A different behavior was observed after exposure to the complexes **1-7**. In term of MCF-7 cells, except for **6**, **HL** complexation increased the antiproliferative activity of the compound, decreasing the proliferation of MCF-7 cells to 89.97%. The highest antiproliferative effect was observed for complexation with CuSO_4_ (complex **2**). In contrast, the **HL** complexation proved to be ineffective for SKBR-3 cells. Only complexation with CuSO_4_ (complex **2**) and Cu(ClO_4_)_2_ (complex **4**) produced a poor increase in **HL**’s antiproliferative activity. In term of A375 cells, **HL** complexation decreased cell proliferation, the best antiproliferative effect being detected for the complex **2** (64.32%). Regarding the proliferation of NCI-H1573 cells, **HL** complexation proved to have a beneficial effect, decreasing cell proliferation. Except for complexes **5** and **7**, all derivatives decreased the proliferation of NCI-H1573 cells by more than 50%, the best result being obtained for **4**_,_ with an inhibition index of 73.58%. Complex **5** decreased cell proliferation with 41.45%, while complex **7** decreased cell proliferation only with 16.46%. In conclusion, the efficiency of complexes depends on the cancer cell line, with NCI-H1573 being the most sensitive.

In case of human leukemia HL-60 cells, the ligand (8-ethyl-2-hydroxytricyclo(7.3.1.0^2,7^) tridecan-13-one-thiosemicarbazone) and its metal complexes (Table 4) were tested as inhibitors of cell proliferation using three concentrations: 0.1, 1.0 and 10 µM.

No effect on HL-60 cell proliferation was noted for the free ligand or salts (data not shown) used in the synthesis of complexes, but as observed, the antiproliferative activity increases by complexation. Compounds **1**–**7** proved to have antiproliferative effects for concentrations higher than 1 µM. This fact is confirmed, especially for copper complex **2**, palladium complex **6** and platinum complex **7**. The antiproliferative activity of the complexes **2**, **6** and **7** at 10 µM is of the same significance as that of doxorubicin, which is utilized in medicinal practice as an antileukemia drug. At this concentration the complexes totally inhibited HL-60 cell proliferation.

The copper complexes **2**, including the bidentate NS ligand and sulfato group, demonstrate an important antiproliferative activity for HL-60 leukemia cells compared to those containing other inner sphere anions. The antiproliferative activity is the highest and the concentration dependence changes from 5.14% to 100%. A similar effect was observed in the case of complexes **6** and **7**. The structures of the tested compounds seemed to be the principal factor influencing the biological activity.

## 3. Experimental Section

### 3.1. General Information

All commercially available reagents and chemicals were of analytical- or reagent-grade purity and used as received. The chemical elemental analysis for the determination of C, H, N was done on a LA-118 microdosimeter (Carlo-Erba, Lakewood, CO, USA). The Cu(II) content has been determined by atomic absorption spectroscopy with a AAS 1N spectrometer (Carl Zeiss Jena, Überlingen, Germany); palladium was determined following the method described by Fries and Getrost [51]. IR spectra were recorded on a Specord-M80 spectrophotometer (Leipzig, Germany) in the 4000–400 cm^−1^ region using KBr pellets. ^1^H-NMR and ^13^C-NMR spectra were recorded at room temperature on a DRX 400 spectrometer (Bruker, Billerica, MA, USA) in DMSO-*d*_6_, using TMS as the internal standard. The complexes were studied by thermogravimetry (TG) in a static air atmosphere, with a sample heating rate of 10 °C/min using a STA 6000 instrument (Perkin Elmer, Waltham, MD, USA). Electronic spectra were recorded using a V-670 spectrophotometer (Jasco, Tokyo, Japan), in diffuse reflectance mode, using MgO dilution matrices. The FAB mass spectra were recorded on a JMS-AX-500 mass spectrometer (JEOL, Tokyo, Japan). GCMS analysis was performed on a GCMS-QP5050A instrument (Shimadzu, Duisburg, Germany). EPR spectra were recorded on polycrystalline powders and DMSO solutions at room temperature and 77 K with a MiniScope MS200 X-band spectrometer (9.3–9.6 GHz) (Magnetech Ltd., Berlin, Germany), connected to a PC equipped with a 100 KHz field modulation unit. The molar conductance of the complexes in dimethylforamide solutions (10^−3^ M), at room temperature, were measured using a Consort type C-533 conductivity instrument (Turnhout, Belgium). The magnetic susceptibility measurements were done at room temperature in the polycrystalline state on a homemade Faraday magnetic balance.

### 3.2. Synthesis

#### 3.2.1. Synthesis of 8-Ethyl-2-hydroxytricyclo[7.3.1.0^2,7^]tridecan-13-one

A flask was flushed with nitrogen and then charged with cyclohexanone (10 mL, 0.947 g, 9.66 mmol). The cyclohexanone was stirred and heated to 70–75 °C under nitrogen. A solution of potassium hydroxide (0.9 g, 16 mmol) in absolute ethanol (15 mL) was added in one portion, and then a solution of propanal (10 mL, 13.6 g, 0.23 mol) in absolute ethanol (14 mL) was added dropwise over a 5 h period while maintaining the reaction mixture at 70–75 °C. The reaction mixture was stirred for an additional 12 h and then cooled to room temperature. The reaction flask was immersed in an ice bath and the mixture was kept at 0 °C for 4 h to complete the crystallization. The colorless crude product was collected by vacuum filtration and washed with cold ether (100 mL). The product was recrystalized from methanol to give 8-ethyl-2-hydroxytricyclo(7.3.1.0^2,7^)tridecan-13-one (1.82 g, 80%), m.p. 167–169 °C, Rf = 0.3 (DCM-petroleum ether = 1:2, silica gel, visualization with KMnO_4_). HRMS [ESI, (+), OrbiTrap] *m*/*z* Calc. for C_15_H_24_O_2_Na [M + Na]^+^ 259.18; Found: 259.1686 [M + Na]^+^.

#### 3.2.2. Synthesis of the 8-Ethyl-2-hydroxytricyclo[7.3.1.0^2,7^]tridecane-13-one-thiosemicarbazone (**HL**)

The ketone 8-ethyl-2-hydroxytricyclo(7.3.1.0^2,7^)tridecan-13-one (0.5 g, 2.12 mmol) was dissolved in ethanol (10 mL). A solution of thiosemicarbazide (0.285 g, 3.13 mmol) in ethanol (7 mL) and water (2.5 mL) was then added. The reaction mixture was refluxed for 24 h and left to cool to room temperature. The resulting precipitate was collected by vacuum filtration. The crude product was recrystallized from a mixture alcohol–water 1:1, to give 0.55 g of the title compound as white crystals, m.p. 201–203 °C, Rf = 0.5 (EtOAc-petroleum ether = 1:2, silica gel). Yield 85%; Anal. Calc. for C_16_H_27_N_3_OS: C, 62.13; H, 8.73; N, 13.59%. Found: C, 62.37; H, 8.52; N, 13.33%. IR (KBr, cm^−1^): υ(N^4′-^H) 3361; υ(N^2′-^H) 3214; υ(C=N^1’^) 1588; υ(C=S + C=N, C=S) 1276, 815; υ(N^1′^–N^2′^) 920. HRMS [ESI, (+)] *m*/*z* Calcd. for C_16_H_27_N_3_OS: 310.1948 [M + H]^+^, 322.1767 [M + Na]^+^, 348.1506 [M + K]^+^; Found: 310.1968 [M + H]^+^, 322.1788 [M + Na]^+^, 348.1529 [M + K]^+^ (Figure S3). ^1^H-NMR (DMSO-*d*_6_, δ, ppm, *J*, Hz): 0.80 (t, 6.9, 3H, H-β); 1.00–2.90 (m, 20H, aliphatic hydrogen); 7.50 (s, 1H, OH,); 9.80 (s, 2H, NH_2_); 10.41 (s, NH); ^13^C-NMR (DMSO-*d*_6_, δ, ppm): 11.89 (C-β); 20.19 (C-6); 20.88(C-11); 20.06 (C-b); 21.42 (C-12); 24.58 (C-4); 26.88 (C-5); 28.35(C-10); 32.67 (C-α); 35.85(C-9); 36.02 (C-3); 42.04 (C-8); 45.07 (C-1); 51.93 (C-7); 75.19 (C-2); 163.88 (C-13); 178.27(C-3′) (Figure S4).

#### 3.2.3. General Procedure for the Preparation of the Metal Complexes

To a solution of 8-ethyl-2-hydroxytricyclo(7.3.1.0^2,7^)tridecan-13-one-thiosemicarbazone (**HL**, 0.620 g, 1 mmol) in methanol (20 mL) was added a solution of the corresponding metal salt in methanol (10 mL). The molar ratio used was 1:2 (M:L) for the copper(II) complexes and 1:1(M:L) for the palladium(II) and platinum(II) complexes. The following metal salts were used: Cu(OAc)_2_·(H_2_O) for complex **1**, CuSO_4_·5H_2_O for complex **2**, Cu(NO_3_)_2_·3H_2_O for complex **3**, Cu(ClO_4_)_2_·6H_2_O for complex **4**, CuCl_2_·2H_2_O for complex **5**, Pd(OAc)_2_ for complex **6**, K_2_(PtCl_4_) for complex **7**. The mixture was stirred for 7 h at 50 °C. The precipitate obtained was filtered, washed with methanol followed by ether and dried *in vacuo*.

(Cu(L)(H_2_O)_2_(OAc)) (**1**). Green solid. Yield: 90%; m.p. 230–233 °C; M. wt.: 466.5; Anal. Calc. for C_18_H_33_CuN_3_O_5_S: C, 46.30; H, 7.07; Cu, 13.61; N, 9.00%. Found: C, 46.53; H, 6.93; Cu, 13.42; N, 8.81%. IR (KBr, cm^−1^): υ(N^4’^-H) 3358; υ(N^2’^-H) 3176; υ(C=N^1’^) 1533; υ(C=S+C=N, C=S) 1267, 802; υ(N^1’^–N^2’^) 934; υ(N^2’^=C) 1617; υ(C-S)600.

(Cu(HL)(H_2_O)_2_(SO_4_)) (**2**). Green solid. Yield: 81%; m.p. 219-221 °C; M. wt.: 505.5; Anal. Calc. for C_16_H_31_CuN_3_O_7_S_2_: C, 38.05; H, 6.14; Cu, 12.58; N, 8.32%. Found: C, 38.27; H, 5.93; Cu, 12.36; N, 8.18%. IR (KBr, cm^−1^): υ(N^4′^-H) 3360; υ(N^2′^-H) 3212; υ(C=N^1′^) 1570; υ(C=S+C=N, C=S) 1265, 803; υ(N^1′^–N^2′^) 927.

(Cu(L)(H_2_O)_2_(NO_3_)) (**3**). Brown solid. Yield: 78%; m.p. 240 °C; M. wt.: 469.5; Anal. Calc. for C_16_H_30_CuN_4_O_6_S: C, 40.89; H, 6.38; Cu, 13.52; N, 11.92%. Found: C, 41.13; H, 6.12; Cu, 13.32; N, 11.72%. IR (KBr, cm^’1^): υ(N^4’^-H) 3362; υ(N^2’^-H) 3184; υ(C=N^1’^) 1543; υ(C=S+C=N, C=S)1251, -; υ (N^1’^–N^2’^) 933; υ(N^2’^=C) 1631; υ(C-S)616.

(Cu(L)(H_2_O)_2_(ClO_4_)) (**4**). Green solid. Yield: 84%; m.p. = 220-222 °C; M. wt.: 507; Anal. Calc. for C_16_H_30_CuN_3_O_7_SCl: C, 37.86; H, 5.91; Cu, 12.52; N, 8.28%. Found: C, 38.21; H, 5.63; Cu, 12. 32; N, 8.18%. IR (KBr, cm^−1^): υ(N^4′^-H) 3357; υ(N^2′^-H) 3198; υ(C=N^1′^) 1570; υ(C=S+C=N, C=S) 1252, 802; υ(N^1′^–N^2′^) 928; υ(N^2′^=C) 1625; υ(C-S) 600.

(Cu(L)_2_(H_2_O)_2_) (**5**). Green solid. Yield: 75%; m.p. 235-237 °C; M. wt.: 715.5; Anal. Calc. for C_32_H_56_CuN_6_O_4_S_2_: C, 53.66; H, 7.82; Cu, 8.87; N, 11.74%. Found: C, 54.03; H, 7.63; Cu, 8.62; N, 11.58%. IR (KBr, cm^−1^): υ(N^4′^-H) 3359; υ(N^2′^-H) 3185; υ(C=N^1′^) 1570; υ(C=S+C=N, C=S) 1256, 804; υ(N^1′^–N^2′^) 931; υ(N^2′^=C) 1626; υ(C-S) 581.

(Pd(L) (OAc)).H_2_O (**6**). Brown solid. Yield: 76 %; m.p. = 264 °C; M. wt.: 491.4; Anal. Calc. for C_18_H_31_PdN_3_O_4_S: C, 43.95; H, 6.30; Pd, 21.65; N, 8.54%. Found: C, 44.13; H, 6.18; Pd, 21.51; N, 8.38%. IR (KBr, cm^−1^): υ(N^4′^-H) 3362; υ(N^2′^-H) 3192; υ(C=N^1′^) 1575; υ(C=S+C=N, C=S) 1247, 804; υ(N^1′^–N^2′^) 929; υ(N^2′^=C) 1613; υ(C-S) 618.

(Pt(L)_2_) (**7**). Yellow solid. Yield: 84 %; m.p. > 300 °C; M. wt.: 811; Anal. Calc. for C_32_H_52_PtN_6_O_2_S_2_: C, 47.34; H, 6.41; N, 10.35%. Found: C, 47.52; H, 6.23; N, 10.20%. IR (KBr, cm^−1^): υ(N^4′^-H) 3358; υ(N^2′^-H) 3190; υ(C=N^1′^) 1578; υ(C=S+C=N, C=S) 1254, 804; υ(N^1′^–N^2′^) 928; υ(N^2′^=C) 1618; υ(C-S) 624.

### 3.3. Antibacterial Activity

The antibacterial activity of complexes and also of their prototype furaciline has been determined in liquid nutritive medium (2% of peptonate broth, pH 7.0), using the successive dilutions method. *Escherichia coli*, *Salmonella enteritidis*, *Staphylococcus aureus*, *Enterococcus faecalis* stems were used as reference culture for *in vitro* experiment. The dissolution of the studied substances in dimethylformamide, microorganisms cultivation, suspension obtaining, determination of minimal inhibition concentration (MIC) and minimal bactericide concentration (MBC) have been carried out according to the method previously reported.

### 3.4. Antifungal Bioassay

Antimycotic properties of the complexes were investigated *in vitro* on laboratory stem *Candida albicans*. The activity has been determined in liquid Sabouroud nutritive environment (pH 6.8). The innoculates were prepared from fungi stems which were harvested during 3–7 days. Their concentration in suspension is (2–4) × 10^6^ colonies forming unities in milliliter. Sowings for yeasts and micelles were incubated at 37 °C during 7 and 14 days, respectively.

### 3.5. Antiproliferative Activity

#### 3.5.1. Preparation of Tested Substances Solutions

Compounds **1**–**7** were dissolved in dimethyl sulfoxide (DMSO; Sigma-Aldrich, Ayrshire, UK) and stored at 2–8 °C. For all experiments, final concentrations of the tested compounds were prepared by diluting the stock solution (10 mM) with culture medium. The final concentration for *in vitro* analysis was 10 µM in case of primary cancer cell lines, while in case of human leukemia HL-60 cells three concentrations (0.1, 1.0 and 10 µM) were used.

#### 3.5.2. Cell Culture

SKBR-3 human breast cancer cell line was cultured in McCoy’s 5a Medium Modified (Sigma Aldrich, Darmstadt, Germany) supplemented with 10% FCS (fetal bovine serum, PromoCell, Heidelberg, Germany), 1% penicillin-streptomycin (Pen/Strep, 10,000 IU/mL; PromoCell). MCF-7 human breast cancer cell line (ATCC, Rockville, MD, USA) was cultured in EMEM (Sigma Aldrich) supplemented with 10% FCS (fetal bovine serum, PromoCell), 1% penicillin-streptomycin (Pen/Strep, 10,000 IU/mL; PromoCell), 1% non-essential amino acids and 1% glutamine (PromoCell). A375 human melanoma cell line was cultured in DMEM containing 15% FCS (fetal bovine serum, PromoCell) and 1% penicillin-streptomycin (Pen/Strep, 10,000 IU/mL; PromoCell). Cells were maintained at an atmosphere of 5% CO_2_ at 37 °C. NCI-H1573 lung adenocarcinoma cell line was cultured in RPMI-1640 Medium (ATCC) supplemented with 10% FCS (fetal bovine serum, PromoCell), 1% penicillin-streptomycin (Pen/Strep, 10,000 IU/mL; PromoCell).

Human promyelocytic leukemia cells HL-60 (ATCC) were routinely grown in suspension in 90% RPMI-1640 (Sigma, St. Louis, MD, USA) containing l-glutamine (2 nM), antibiotics (100 IU penicillin/mL, 100 µg streptomycin/mL) and supplemented with 10% (*v*/*v*) foetal bovine serum (FBS), in a 5% CO_2_ humidified atmosphere at 37 °C. Cells were currently maintained twice a week by diluting the cells in RPMI 1640 medium containing 10% FBS.

#### 3.5.3. Cell Proliferation Assays

##### Alamar Blue *in Vitro* Analysis

Human breast cancer cell lines (SKBR-3 and MCF-7), lung adenocarcinoma cell line (NCI-H1573) and human melanoma cell line (A375) were seeded onto a 96-well microplate (5000 cells/plate) and let overnight to attach to the bottom of the well. Next day, a volume of 150 μL of the tested substances dissolved into the culture medium were added. After an incubation of 48 h, 15 μL of the Alamar blue (BioSource, Camarillo, CA, USA) solution was added and the cells were incubated at 37 °C. After another 10 h, a microplate reader was used to spectrophotometrically analyze the samples. The wave lengths used were 570 nm, 600 nm respectively. Wells with untreated cells were used as controls. The highest concentration of DMSO (0.1%) used to prepare stock solutions of the tested substances did not have any significant effect on cell proliferation.

Cells viability was calculated using the formula:

100 − {((ε_OX_)λ_2_ Aλ_1_ − (ε_OX_)λ_1_ Aλ_2_ of test agent dilution)/((ε_OX_)λ_2_ A°λ_1_ − (ε_OX_)λ_1_ A°λ_2_ of untreated positive growth control)} × 100 (1)

where ε_OX_ = molar extinction coefficient of Alamar blue oxidized form (BLUE); A = absorbance of test wells; A° = absorbance of positive growth control well ( cells without tested compounds); λ_1_ = 570 nm, λ_2_ = 600 nm. All measurements were performed in triplicate and data were presented as mean ± standard deviation.

##### MTS Cell Proliferation Assay

The cell proliferation assay was performed using 3-(4,5-dimethylthiazol-2-yl)-5-(3-carboxymethoxyphenyl)2-(4-sulfophenyl)-2*H*-tetrazolium (MTS) (Cell Titer 96 Aqueous, Promega, Madison, WI, USA), which allowed us to measure the number of viable cells. In brief, triplicate cultures of 1 × 10^4^ cells in a total of 100 µL medium in 96-well microtiter plates (Becton Dickinson and Company, Lincoln Park, NJ, USA) were incubated at 37 °C, 5% CO_2_. Final concentrations of the tested compounds and doxorubicin (Novapharm, Toronto, ON, Canada) were added to each well and incubated for 3 days. Following each treatment, 12 µL MTS was added to each well and incubated for 4 h. MTS is converted to water-soluble colored formazan by a dehydrogenase enzyme present in metabolically active cells. Subsequently, the plates were read at 490 nm using a microplate reader (Molecular Devices, Sunnyvale, CA, Canada).

## 4. Conclusions

The coordination ability of the thiosemicarbazone 8-ethyl-2-hydroxytricyclo(7.3.1.0^2,7^)tridecan-13-one-thiosemicarbazone (**HL**) has been proved in complexation reactions with Cu(II), Pd(II) and Pt(II) ions. The physico-chemical analyses confirmed the composition and the structures of the newly obtained complexes. Independent of the metal salt anion used, the ligand acts as a mononegative bidentate species through the thioenolic sulphur and the azomethinic nitrogen, except for in complex **2**, in which the ligand acts as neutral bidentate.

The EPR spectra of the copper (II) complexes **1**–**5** in DMSO solution confirmed the new structures. The EPR parameters g_//_, g_⊥_, A_//_ and the energies of d–d transitions were used to evaluate the bonding parameters. The orbital reduction factors indicate the presence of out-of-plane π-bonding for complexes **2**, **4**, **5** and of some in-plane π-bonding for the complexes **1** and **3**.

The ligand and the metal complexes **1**–**7** have been screened for their *in vitro* antiproliferative activity against NCI-H1573 lung adenocarcinoma, SKBR-3 human breast, MCF-7 human breast, A375 human melanoma cancer cells and HL-60 human promyelocytic leukemia cells and for their *in vitro* antimicrobial activity against *Escherichia coli*, *Salmonella enteritidis*, *Staphylococcus aureus*, *Enterococcus faecalis* and *Candida albicans.*

The highest antiproliferative activity of all compounds was for the NCI-H1573 lung adenocarcinoma and MCF-7 breast cancer cell lines, while SKBR-3 breast cancer cells seems to be more resistant to treatment. Results of antiproliferative activity towards HL-60 cells indicated that the ligand alone has no cytotoxic effects at the three tested concentrations. The complexes **2**, **6** and **7** significantly reduced the malignant HL-60 cell growth.

The quantitative antimicrobial activity test results proved that both the ligand and complex combinations have specific antimicrobial activity, depending on the microbial species tested.

We believe that the antiproliferative activity is influenced by the type of chemical bond existing in-plane and out-of-plane of the molecule. Good results were recorded for compounds showing K_//_ > K_⊥._ These promising results are encouraging for further research in this field. Our continued, detailed studies of the toxicity of these compounds, as well as mechanism of action are in process, which could be helpful in designing more potent antitumor agents for therapeutic use.

## Figures and Tables

**Table 1:** Electronic spectra (cm^−1^) and magnetic moment (BM) of the complexes **1**–**7**.

Metal Complex Molecular Formula		Transitions d–d (cm^−1^)		µ_eff_ (BM)	Geometry
(Cu(L)(H_2_O)_2_(OAc)) (**1**)	^2^B_1g_→^2^A_1g_ -	^2^B_1g_→^2^B_2g_ 13,570	^2^B_1g_→^2^E_g_ 20,000	1.99	Octahedral distorted
(Cu(HL)(H_2_O)_2_(SO_4_)) (**2**)	^2^B_1g_→^2^A_1g_ -	^2^B_1g_→^2^B_2g_ 13,570	^2^B_1g_→^2^E_g_ 20,000	1.83	Octahedral distorted
(Cu(L)(H_2_O)_2_(NO_3_)) (**3**)	^2^B_1g_→^2^A_1g_ -	^2^B_1g_→^2^B_2g_ 12,690	^2^B_1g_→^2^E_g_ 20,000	1.69	Square-pyramidal
(Cu(L)(H_2_O)_2_(ClO_4_)) (**4**)	^2^B_1g_→^2^A_1g_ -	^2^B_1g_→^2^B_2g_ 13,570	^2^B_1g_→^2^E_g_ 20,100	1.88	Octahedral distorted
(Cu(L)_2_(H_2_O)_2_) (**5**)	^2^B_1g_→^2^A_1g_ -	^2^B_1g_→^2^B_2g_ 12,550	^2^B_1g_→^2^E_g_ 20,000	1.97	Octahedral distorted
(Pd(L)(OAc))(H_2_O) (**6**)	^1^A_1g_→^1^B_1g_ -	^1^A_1g_→^1^A_2g_ 22,220	^1^A_1g_→^1^E_g_ -	*	Square-planar
(Pt(L)_2_) (**7**)	^1^A_1g_→^1^B_1g_ -	^1^A_1g_→^1^A_2g_ 23,250	^1^A_1g_→^1^E_g_ -	*	Square-planar

* diamagnetic.

**Table 2:** EPR spectral parameters of the Cu(II) complexes **1**–**5**.

	1	2	3	4	5
Polycrystalline (298 K)					
g_//_	2.404	2.371	2.208	2.409 (g_3_)	2.237
g_⊥_	2.087	2.103	2.041	2.193 (g_2_)	2.065
				2.011 (g_1_)	
DMSO (77K)					
g_//_	2.394	2.400	2.399	2.399	2.400
g_⊥_	2.082	2.088	2.076	2.078	2.098
A_//_	117.4	118.0	119.2	118.0	119.0
α^2^	0.789	0.838	0.802	0.792	0.831
β^2^	0.989	0.998	0.890	0.996	0.978
δ^2^	0.992	0.879	0.973	0.969	0.952
K_//_	0.780	0.841	0.717	0.769	0.787
K_⊥_	0.791	0.569	0.785	0.758	0.753

**Table 3:** Antibacterial and antifungal activities of ligand (**HL)** and complexes **1**–**7** as MIC ^a^/MBC ^b^ values (mg/mL).

Compounds	*E. coli* (G−)		*S. enteritidis* (G−)		*S. aureus* (G+)		*E. faecalis* (G+)		*C. albicans*	
	MIC	MBC	MIC	MBC	MIC	MBC	MIC	MBC	MIC	MBC
C_16_H_27_N_3_OS (**HL**)	0.5	>10.0	>10.0	>10.0	0.5	>10.0	>10.0	>10.0	0.5	>10.0
(Cu(L)(H_2_O)_2_(OAc)) (**1**)	>10.0	>10.0	0.5	>10.0	>10.0	>10.0	>10.0	>10.0	>10.0	>10.0
(Cu(HL)(H_2_O)_2_(SO_4_)) (**2**)	0.25	>10.0	0.25	0.12	0.12	0.12	0.12	0.12	0.12	0.12
(Cu(L)(H_2_O)_2_(NO_3_)) (**3**)	>10.0	>10.0	>10.0	>10.0	0.12	0.25	0.12	0.12	0.5	0.5
(Cu(L)(H_2_O)_2_(ClO_4_)) (**4**)	0.5	>10.0	0.5	>10.0	0.12	>10.0	0.12	>10.0	0.12	0.12
(Cu(L)_2_(H_2_O)_2_) (**5**)	>10.0	>10.0	0.5	>10.0	0.12	>10.0	0.12	>10.0	0.12	0.12
(Pd(L)(OAc))(H_2_O) (**6**)	>10.0	>10.0	0.5	>10.0	0.5	>10.0	>10.0	>10.0	>0.5	>0.5
(Pt(L)_2_) (**7**)	>10.0	>10.0	0.5	>10.0	>10.0	>10.0	0.12	>10.0	>10.0	>10.0
Cu(OAc)_2_·H_2_O	0.5	-	1.024	-	1.024	-	0.5	-	-	-
CuSO_4_·5H_2_O	0.5	-	1.024	-	0.5	-	0.5	-	1.024	-
Cu(NO_3_)_2_·3 H_2_O	1.024	-	1.024	-	1.024	-	1.024	-	-	-
Cu(ClO_4_)_2_·6 H_2_O	1.024	-	1.024	-	1.024	-	0.5	-		-
CuCl_2_·2H_2_O	1.024	-	0.5	-	0.5	-	0.5	-	-	-
Pd(OAc)_2_	1.024	-	1.024	-	1.024	-	0.5	-		-
K_2_(PtCl_4_)	1.024	-	1.024	-	1.024	-	0.5	-		-
Furacillinum	0.018	0.037	0.009	0.009	0.009	0.009	0.037	0.037	-	-
Nystatine	-	-	-	-	-	-	-	-	0.08	0.08

*E. coli* (*Escherichia coli*, ATCC 25922); *S. enteritidis* (*Salmonella enteritidis*); *S. aureus* (*Staphylococcus aureus*, ATCC 25923); *E. faecalis (Enterococcus faecalis)*; *C. albicans* (*Candida albicans*). ^a^ MIC–minimum inhibitory concentration. ^b^ MBC—minimum bactericide concentration. G(−): Gram-negative bacteria; G(+): Gram-positive bacteria.

**Table 4:** Antiproliferative activity of ligand and metal complexes on human leukemia HL-60 cells at three concentrations ^a^.

Compound	Inhibition of Cell Proliferation %			IC_50_ (µM) ^b^
	10 µM	1 µM	0.1 µM	
C_16_H_27_N_3_OS (**HL**)	−2.90	−5.72	0.31	-
(Cu(L)(H_2_O)_2_(OAc)) (**1**)	21.97	−2.11	−2.58	≥10
(Cu(HL)(H_2_O)_2_(SO_4_)) (**2**)	100.00	5.14	-5.25	1.6 ± 3
(Cu(L)(H_2_O)_2_(NO_3_)) (**3**)	63.91	8.12	1.35	6.4 ± 5
(Cu(L)(H_2_O)_2_(ClO_4_)) (**4**)	48.94	6.48	1.13	6.5 ± 4
(Cu(L)_2_(H_2_O)_2_) (**5**)	43.43	8.73	7.20	14.2 ± 3
(Pd(L)(OAc))(H_2_O) (**6**)	100.00	−22.99	−3.85	6.5 ± 4
(Pt(L)_2_) (**7**)	100.00	−10.32	−8.13	6.4 ± 2
DOXO ^c^	91.94	94.55	77.00	≤0.1

^a^ SEM < ± 4% of a single experiment in triplicate. ^b^ The IC_50_ value was defined as the concentration at which 50% survival of cells was observed. ^c^ DOXO (doxorubicin) used as a positive control.

## Supplementary Materials

Supplementary materials can be accessed at: http://www.mdpi.com/1420-3049/21/5/674/s1.
