# Supplementary material for: Synthesis and Characterization of Novel Cu(II), Pd(II) and Pt(II) Complexes with 8-Ethyl-2-hydroxytricyclo(7.3.1.02,7)tridecan-13-one-thiosemicarbazone: Antimicrobial and in Vitro Antiproliferative Activity
Source: Molecules. 2016 May 21;21(5):674. doi: 10.3390/molecules21050674 (PMC6273217; doi:10.3390/molecules21050674)
Supplement: Supplementary file 1 [file molecules-21-00674-s001.pdf]

# Supplementary Materials: Synthesis and Characterization of Novel Cu(II), Pd(II) and Pt(II) Complexes with 8-Ethyl-2-hydroxytricyclo(7.3.1.0$^{2,7}$)tridecan-13-one-thiosemicarbazone Antimicrobial and *In vitro* Antiproliferative Activity

**Elena Pahonțu, Codruța Paraschivescu, Diana-Carolina Ilieș, Donald Poirier, Camelia Oprean, Virgil Păunescu, Aurelian Gulea, Tudor Roșu and Ovidiu Bratu**

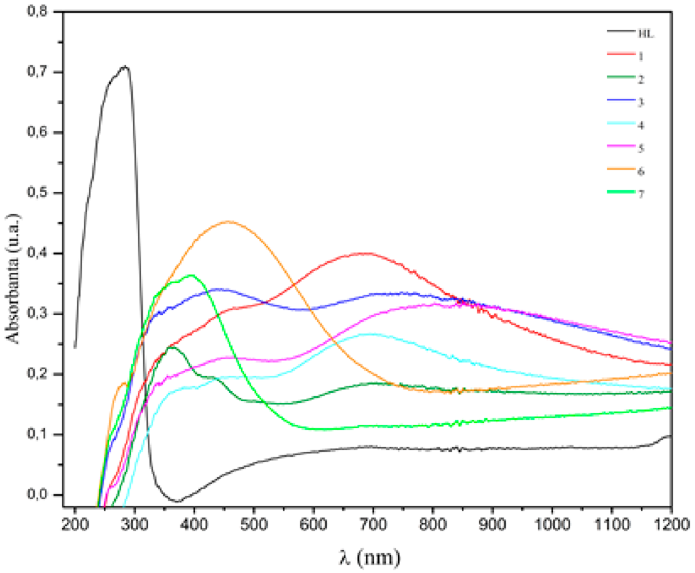


**Figure S1.** Electronic spectra of complexes **1**–**7**.

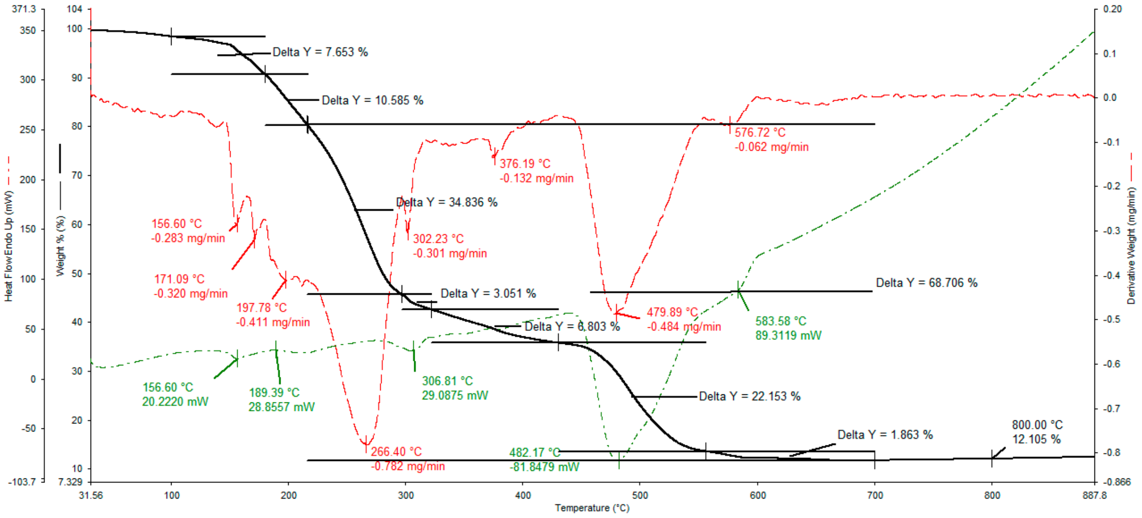


**(a)**

**Figure S2.** *Cont.*

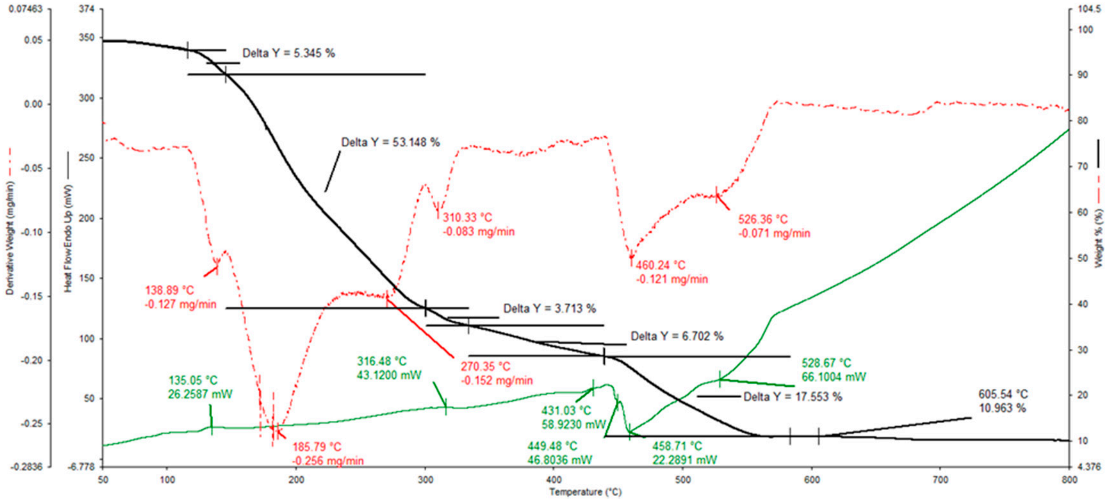


(**b**)

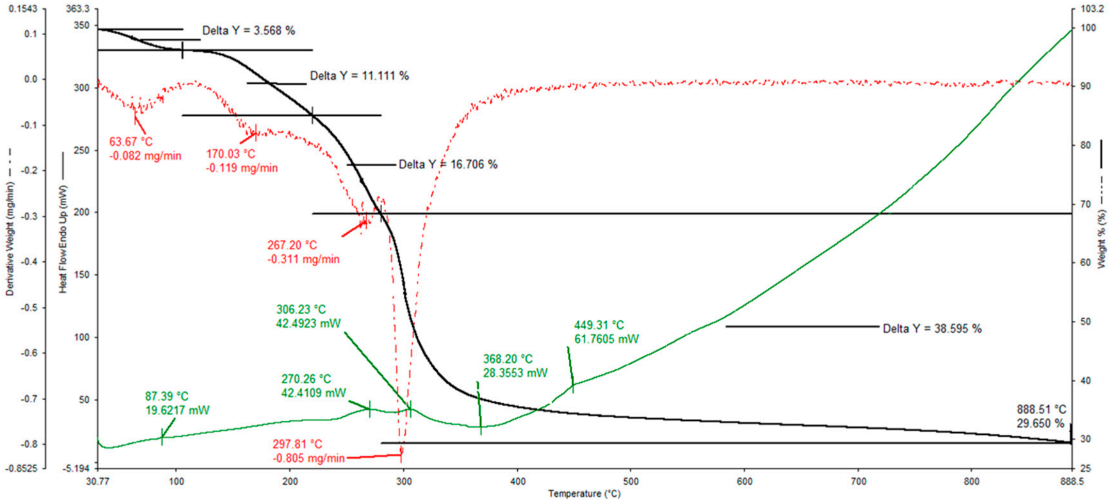


(**c**)

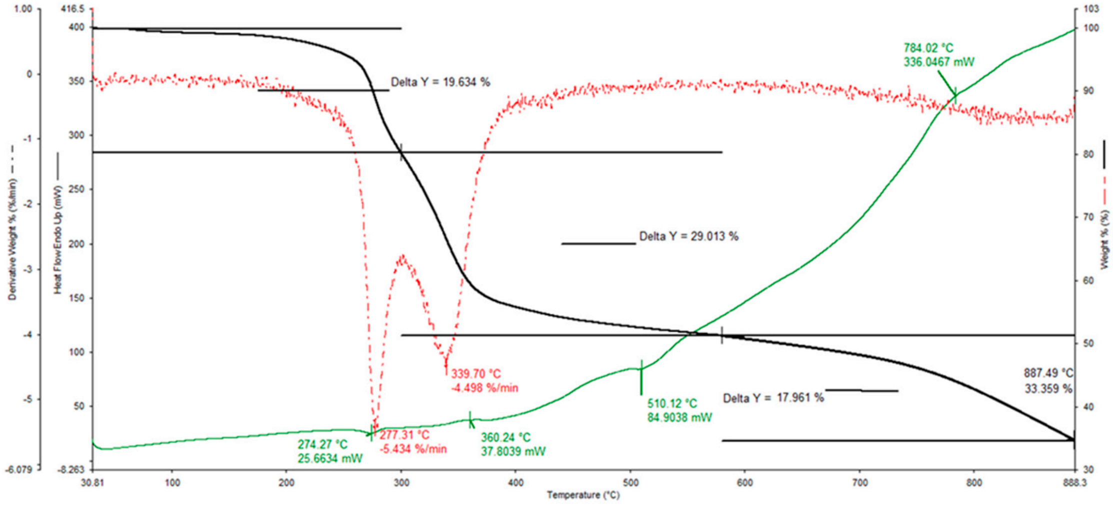


(**d**)

**Figure S2.** Thermogravimetric analysis of complexes (**a**) **1**, (**b**) **5**, (**c**) **6** and (**d**) **7**.

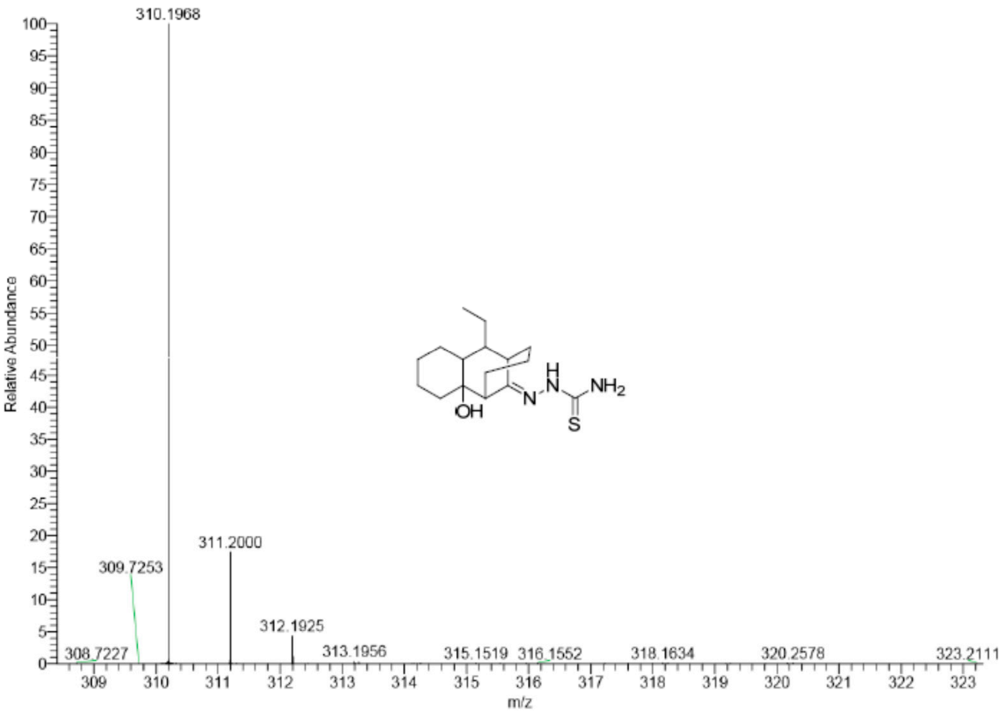


**Figure S3.** The mass spectrum of ligand **HL**.

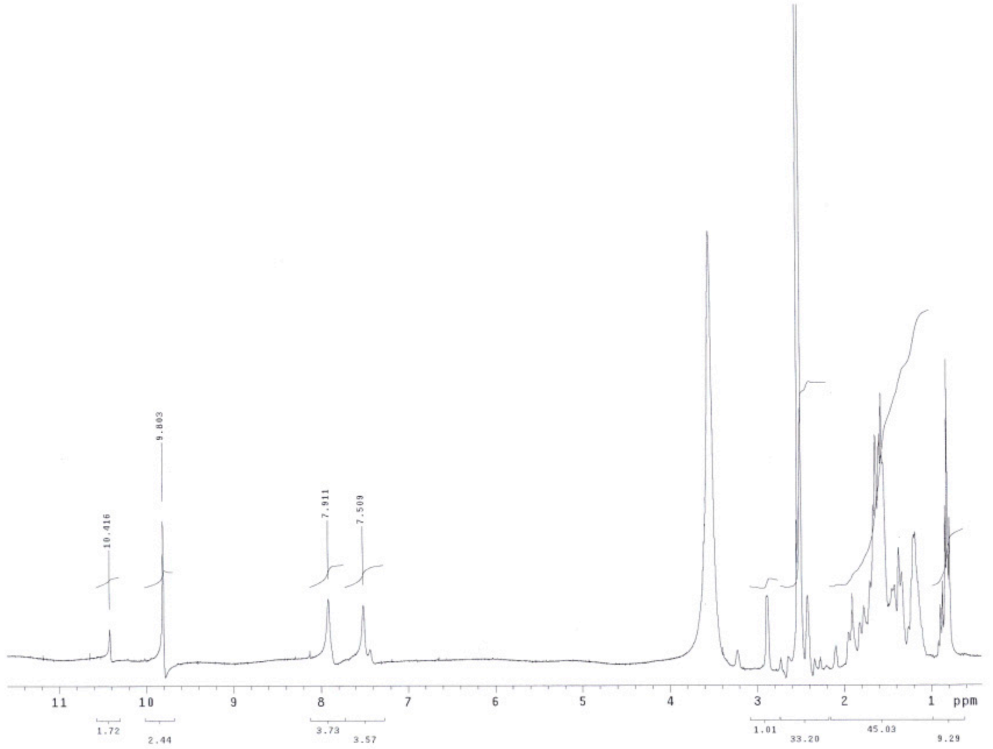


(**a**)

**Figure S4.** *Cont.*

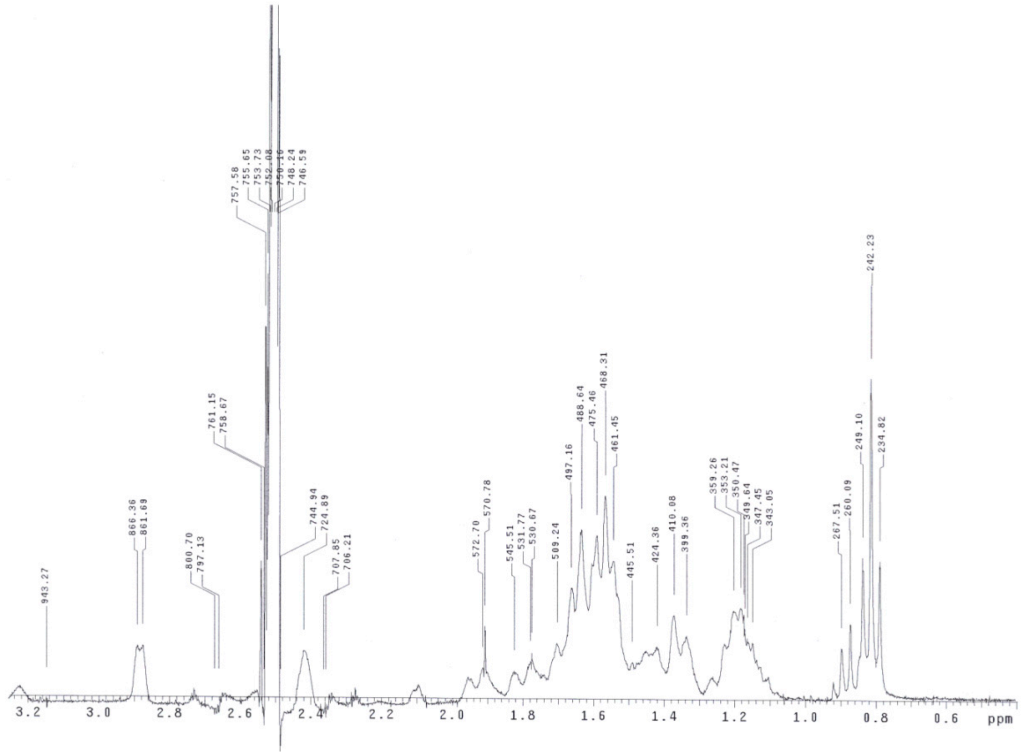


(**b**)

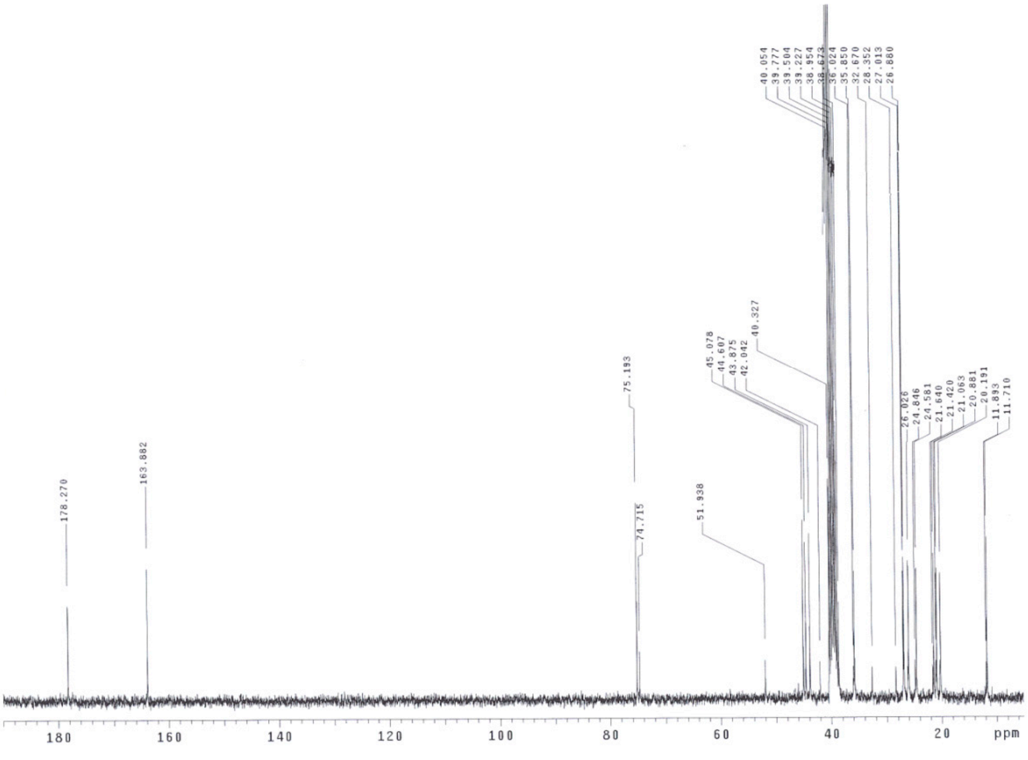


(**c**)

**Figure S4.** The spectra $^{1}H$-NMR (**a**,**b**) and $^{13}C$-NMR (**c**) of ligand (**HL**).

**Table S1.** FAB mass spectral data of complexes **1**–**7**.

| Molecular Formula | Mw (g/mol) | Molecular Ion Peak $[M]^+$ | The Peaks Due to Complex Fragmentation | | | |
|---|---|---|---|---|---|---|
| $(Cu(L)(H_2O)_2(OAc))$ (**1**) | 466.5 | 372.5 | 201.1 | 216.1 | 259.1 | 292.1 |
| $(Cu(HL)(H_2O)_2(SO_4))$ (**2**) | 505.5 | 374.2 | 201.1 | 219.1 | 259.1 | 275.1 |
| $(Cu(L)(H_2O)_2(NO_3))$ (**3**) | 469.5 | 371.5 | 188.1 | 219.1 | 254.2 | 292.2 |
| $(Cu(L)(H_2O)_2(ClO_4))$ (**4**) | 507 | 372.5 | 202.3 | 217.0 | 253.2 | 293.1 |
| $(Cu(L)_2(H_2O)_2)$ (**5**) | 715.5 | 681.3 | 340.2 | 404.1 | 473.3 | 593.1 |
| $(Pd(L)(OAc))(H_2O)$ (**6**) | 491.4 | 474.5 | 281.1 | 341.2 | 370.1 | 416.2 |
| （$Pt(L)_2$） (**7**) | 811 | 794.1 | 501.1 | 587.1 | 616.2 | 705.1 |

**Table S2.**The numerical values of the Alamar blue analysis (with standard deviation).

| Compound | HL | 1 | 2 | 3 | 4 | 5 | 6 | 7 |
|---|---|---|---|---|---|---|---|---|
| | | | | **A375** | | | | |
| Viability % | 99.819 | 81.482 | 35.676 | 47.108 | 69.523 | 76.913 | 89.080 | 96.075 |
| * | 2.315 | 2.350 | 11.009 | 3.001 | 7.861 | 2.260 | 5.913 | 4.492 |
| | | | | **MCF-7** | | | | |
| Viability % | 71.421 | 17.292 | 10.025 | 12.847 | 12.698 | 36.891 | 74.421 | 69.267 |
| * | 5.553 | 0.270 | 0.141 | 0.515 | 1.604 | 0.425 | 3.421 | 1.063 |
| | | | | **SKBR-3** | | | | |
| Viability % | 75.130 | 80.582 | 72.626 | 94.081 | 67.155 | 92.974 | 90.713 | 90.896 |
| * | 13.479 | 1.277 | 5.419 | 7.123 | 5.163 | 8.702 | 11.107 | 4.803 |
| | | | | **NCI-H1573** | | | | |
| Viability % | 87.63 | 29.61 | 31.33 | 43.35 | 26.42 | 58.55 | 38.85 | 83.54 |
| * | 4.72 | 2.76 | 2.70 | 4.72 | 2.88 | 3.18 | 1.75 | 4.35 |

*standard deviation. The values are presented as percent of control (100% viability).
